# Health-related quality of life (HRQL) for individuals with self-reported chronic physical and/or mental health conditions: panel survey of an adult sample in the United States

**DOI:** 10.1186/1477-7525-10-154

**Published:** 2012-12-19

**Authors:** Martha Bayliss, Regina Rendas-Baum, Michelle K White, Mark Maruish, Jakob Bjorner, Sandra L Tunis

**Affiliations:** 1 QualityMetric, Inc., part of OptumInsight, 24 Albion Road, Building 400, Lincoln, RI, 02865, USA

## Abstract

**Background:**

In the US, approximately 53% of adults have *at least* one chronic condition. Comorbid physical and mental health conditions often have an incremental negative impact on health-related quality of life (HRQL). Primary study objectives were to quantify the impact on HRQL of a) ≥ 1 physical condition , b) ≥ 1 comorbid mental health conditions added to a physical one, c) ≥ 1 mental health condition, and d) ≥ 1 comorbid physical conditions added to at least one related to mental health. Decrements were based on a “Healthy” reference group reporting no chronic conditions.

**Methods:**

Participants were sampled (n = 3877) from the US adult population as part of a 2009 normative survey. Demographics, number/ type of chronic conditions, and HRQL data were self-reported. HRQL was defined through SF-36v2® Physical Component Summary (PCS) scores and Mental Component Summary (MCS) scores. Participant “morbidity” groupings included *Healthy*; *Physical Health Condition* only, *Mental Health Condition* only, and *Physical and Mental Health (Comorbid).* PCS and MCS scores were also analyzed by physical disease clusters (e.g., cardiovascular, gastrointestinal). Multivariate regression models were used for all analyses.

**Results:**

81% of participants were Caucasian; 9% African American. Males and females were about equally represented; 63% were ≥ 45 years old. The average number of reported chronic conditions was 2.4 (SD = 2.4). Relative to the *Healthy* group, the *Physical Condition* group scored 6.4 (males) and 7.5 (females) points lower on PCS. The addition of a comorbid *mental health* condition resulted in a total reduction of 11 points in PCS and 15 points in MCS. Compared to the *Healthy* group, ≥ 1 *mental health* conditions was associated with MCS decrements of 11–12 points. A *physical* comorbidity led to additional decrements of 3–4 points for MCS, with a total of 15 points. Incremental HRQL burden defined by both MCS and PCS scores was relatively similar across the 5 defined physical disease clusters.

**Conclusion:**

Results provide quantitative information for US adults on specific PCS and MCS score decrements associated with a comorbid condition related to mental health, as well as a comorbid condition related to physical health.

## Background

### Chronic conditions in the United States

The humanistic and economic burden of chronic health conditions has been well-documented in the United States (US) and elsewhere. In the US, approximately half (53%) of the adult population has *at least* one chronic condition related to physical or mental health [1]. Moreover, 7% of adults 45–54 years of age, and 37% of those 75 years or older report having three or more [2]. Although most chronic conditions can be controlled through treatment and long-term management, they are the primary reason for seeking medical care and the leading contributors to disability and mortality [3].

Certain chronic conditions related to physical health are of particular concern due to their comparatively high prevalence, as well as their link to additional morbidities and negative effects on daily functioning. For example, the US prevalence of diabetes for those at least 20 years of age is approximately 11% [4], and has been linked to a variety of cardiovascular and other types of complications [4,5]. The overall prevalence of coronary heart disease (CHD) for US adults is 6-7%, and remains a leading cause of morbidity and mortality [6]. Finally, about one-third of adults over age 65 experience chronic symptomatic osteoarthritis, the leading cause of disability in older adults [7].

Several chronic conditions related to mental health are also comparatively prevalent in the US adult population. The lifetime and 12-month prevalence of any anxiety disorder is 29% and 18%, respectively. The prevalence of major depression is 17% (lifetime) and 7% (12-month) [8,9].

### Physical and mental health comorbid chronic conditions

A large body of research documents the co-occurrence of a *specific chronic physical condition* (e.g., congestive heart failure (CHF), diabetes) and one related to *mental health* such as depression or anxiety [10-13]. Certain chronic conditions frequently precipitate and/or exacerbate depressive symptoms [14,15]. Conversely, depression can lead to- or worsen conditions in the *physical* domain such as asthma, cardiovascular disease, and diabetes [15,16]. Comorbid physical and mental health conditions can show a combined negative impact on disease-related outcomes [7,15].

To illustrate, among participants with type 2 diabetes (n = 249) in a UK study, those with anxiety and/or depression reported significantly more days off work, as well as decreased adherence to diabetes medication (p < .05 for both outcomes) [17]. In another study of veterans with type 2 diabetes, the presence of comorbid depression and/or alcohol/drug abuse had a significant and negative impact on diabetes-specific outcomes, as well as all-cause mortality [18].

### Chronic conditions and Health-Related Quality of Life (HRQL)

Studies assessing the burden of chronic physical and mental health conditions increasingly include the negative impact on an individual’s health-related quality of life (HRQL), commonly defined as everyday functioning and well-being [19-22]. One recent study of US adults (n = 4833) retrospectively compared self-reported burden of chronic conditions related to *mental health,* to those of three related to *physical health* (back/neck problems, hypertension, diabetes) [23]. Burden was defined by the number of days in the previous 30 when a person indicates that activities are limited due to physical or mental health difficulties [24-26].

Controlling for demographic variables, adults with *mental health* conditions reported 6.8 fewer healthy days/ month compared to those without these conditions (p < 0.001). Among adults with one of the 3 *physical* conditions, the mean number of additional unhealthy days (versus those with none of these physical conditions) ranged from 1.0 (for hypertension, n.s.) to 3.6 (back and neck problems, p < 0.001). When compared directly, *mental health* conditions were associated with significantly lower HRQL than were any of the 3 related to *physical health* (p = 0.002 to 0.053).

Aggregating different chronic conditions, Rothrock et al. [27] examined the association of *number of conditions* and HRQL. Study participants (n = 21,133) reported the presence or absence of 24 possible chronic health conditions, and whether or not their activity was limited by each. Across all 5 health-status domains assessed (physical function, fatigue, pain, emotional distress, and social function), the presence of a chronic condition was associated with poorer scores relative to no chronic condition. For those with ≥ 2 morbidities, decrements in HRQL were significantly more pronounced when compared to those with no condition, or to those with a single condition (p < .0001).

Finally, a large recently published retrospective study of US adults [24] was designed to examine HRQL based on both *number* and *type* of chronic conditions. From Behavioral Risk Factor Surveillance System (BRFSS) 2007 data [28], 57% of the sample (n = 430,912) reported at least 1 chronic condition; with arthritis, obesity, and hypertension the most prevalent.

With adjustments for relevant variables (age, sex, race/ethnicity, education, income, employment, marital status, health insurance, smoking, alcohol consumption, physical activity), those with ≥ 3 conditions were more likely to report fair or poor health (for general health, mental distress, physical distress, activity limitations) compared to those reporting 1 or 2 chronic conditions. Compared to other diseases, *cardiovascular* conditions or *diabetes* had the largest impact on HRQL. Individuals with one of these two conditions were 7–8 times more likely (than were those with no conditions) to report poor or fair HRQL.

### Use of the SF-36 health survey in assessing HRQL impact of chronic conditions

Many studies [22,29-32] have examined the association of chronic conditions and HRQL as defined by the Physical Component Summary (PCS) and the Mental Component Summary (MCS) scores of the widely-used SF-36® Health Survey [33-35]. The domains of physical functioning (PF), role-physical (RP), bodily pain (BP), and general health (GH) correlate most highly with the PCS, while the mental health (MH), role-emotional (RE), vitality (VT) and social functioning (SF) correlate most highly with the MCS.

A systematic review of published studies (1990–2003) examining multi-morbidity and HRQL among individuals in primary care treatment settings showed that 22 of the 30 studies examined used the SF-36 as the main tool to assess HRQL [30]. Although use of a standard tool greatly enhanced the comparability of results across studies, a common methodological limitation noted in the review was the failure to evaluate the effect of mental health comorbidity.

A more recent study [29] reported that the negative impact of *mental health* conditions on PCS scores was greater than the negative impact of various *physical health* conditions on MCS. Given the relatively high prevalence of several mental health conditions in the US, the cumulative impact of *physical* and *mental health* comorbidity on *both* Physical and Mental Health domains of HRQL warrants further examination.

### Study objectives

The goal of this study was to examine the burden of self-reported chronic *physical and/or mental health conditions* on HRQL*.* Primary objectives were to quantify the HRQL impact (compared to a “Healthy” group reporting no chronic conditions) of a) at least 1 physical condition, , b) 1 or more comorbid mental health conditions added to a physical one, c) at least 1 mental health condition, and d) 1 or more comorbid physical conditions added to a mental health one. A secondary objective was to examine the extent to which patterns of HRQL burden noted overall were similar across specific organ-system domains or Physical “Disease Clusters”.

## Methods

### Participant sample and data collection

The current study was part of a QualityMetric 2009 Norming Study conducted in 2009 [35]. Study participants were sampled from the US general population of (non-institutionalized) adults aged 18 or older who were fluent in English. They were recruited through the Knowledge Panel® maintained by Knowledge Networks [36] (now GfK Custom Research, LLC), using a validated method of probability sampling of residential addresses covering approximately 97% of US households. All data were based on self-report and were collected via the Internet between June and October of 2009. Demographic information obtained included age, gender, race, education, and employment and marital status.

### Assessment of chronic conditions

Using a chronic condition checklist, participants reported whether they had ever been told by a doctor or other health care professional that they had any of 26 possible chronic conditions. The 23 chronic *physical* conditions included those related to the cardiovascular, endocrine, respiratory, musculoskeletal, and gastrointestinal domains, as well as other conditions such as migraine headaches, and cancer. The three chronic *mental health* conditions in the checklist were clinical depression, anxiety, and an alcohol or other substance use disorder.

### Assessment of Health-Related Quality of Life (HRQL)

Participants completed the SF-36v2® Health Survey (SF-36v2), a widely-used generic measure of HRQL [35]. The SF-36v2 has been extensively validated, and has been used to assess and compare disease burden, as well as treatment effectiveness in a very large number of chronic health conditions, including those related to both physical and mental health [29,30]. The eight domains derived from 36 item responses were aggregated into the Physical Component Summary (PCS) and the Mental Component Summary (MCS) scores.

Based on a large sample of adults in the US, the two extensively documented component summary measures are each scored on the *T*-score metric, with a mean of 50.0 (and a standard deviation of 10.0). As with the prior version of the SF-36, one important aspect of the SF-36v2 construct validation procedures included known-groups comparisons [34,35]. These analyses demonstrated the ability of the PCS and MCS measures to discriminate groups of individuals with only a physical or a mental health condition from a “healthy” group and from groups with comorbid (physical and mental health) conditions [35].

### Statistical analyses

Based on checklist responses, four “morbidity” groups were created for comparisons relevant to the primary study objectives:

1. *Healthy:* no reported chronic conditions related to physical or mental health.

2. *Physical Health Condition:* 1 or more chronic physical conditions, but none related to mental health.

3. *Mental Health Condition:* 1 or more chronic mental health conditions, but none related to physical health.

4. *Physical and Mental Health (Comorbid) Condition:* one or more chronic physical conditions *and* one or more related to mental health.

The mean PCS and MCS scores of participants in the a) *Physical Health Condition* group, and b) *Mental Health Condition* group were separately compared to scores of those in the *Healthy* group to evaluate the HRQL impact of having one or more chronic physical conditions or one or more related to mental health. Further, a comparison of the a) *Physical Health Condition* group and b) *Physical and Mental Health Condition (Comorbidity)* group was made to evaluate the impact of ≥ 1 mental health conditions in addition to a ≥ 1 physical conditions. Finally, a comparison of the a) *Mental Health Condition* group and b) *Physical and Mental Health Condition (Comorbidity)* group was conducted to evaluate the HRQL impact of ≥ 1 physical conditions in addition to a having ≥ 1 mental health conditions.

Mean PCS and MCS scores for each of the defined “morbidity” groups were estimated using multivariate regression models with group membership, age, and an interaction term between group and age as independent variables. Mean PCS and MCS values were estimated by setting age at the mean of the entire sample of Normative Study participants (51 years). This was done to ensure that PCS and MCS were estimated at the same value of age for all morbidity groups and for both males and females, so as to ensure a comparability of results. Models were run to obtain comparisons separately for males and females.

### Secondary objective: analyses by physical disease cluster

To address the secondary objective, a subset of physical conditions was categorized into one of five *physical disease clusters* listed below. Participants were then grouped by Disease Cluster. This clustering approach was used to overcome cumbersome analytics and sample requirements involved with studying individual conditions [29,37,38].

1. *Cardiovascular Cluster:* heart attack in past year; congestive heart failure (CHF); angina or coronary artery disease; other heart conditions

2. *Gastrointestinal Cluster:* liver disease; kidney disease; stomach disease; irritable bowel syndrome (IBS)

3. *Endocrine Cluster:* type 1 diabetes, type 2 diabetes

4. *Musculoskeletal Cluster:* rheumatoid arthritis (RA); osteoarthritis; degenerative arthritis; osteoporosis

5. *Respiratory Cluster:* chronic obstructive pulmonary disease (COPD)

The incremental PCS burden of a comorbid mental health condition was evaluated by comparing, within each disease cluster, the mean PCS scores of participants with no mental health conditions to the mean PCS scores of those who also had at least 1 mental health condition. Conversely, the incremental MCS burden of a physical comorbidity was evaluated by comparing the mean MCS scores of participants reporting ≥ 1 mental health condition but no physical health conditions, to the mean MCS scores of those reporting ≥ 1 mental health conditions and ≥ 1 condition from each “Physical Disease Cluster” specified above.

## Results

### Participant characteristics

As shown in Table 1, the majority of the 3,877 study participants were Caucasian (81%), and 9% were African American. Males (49%) and females (51%) were represented almost equally. Approximately equal percentages reported their education level as high school completion (30%); some college (31%); or a bachelor’s degree or higher (31%). More than half were working (53%) and almost one fourth (24%) reported being retired. Fifty-eight percent reported being married or living with a partner, 15% being separated or divorced, and 20% never having been married.

**Table 1:** Sample characteristics by “Morbidity Group”

	**Healthy (1,505)**	**Physical (1,522)**	**Mental (236)**	**Physical & Mental (614)**	**Total sample (3,877)**
**Mean (SD)**					
**Age**	44.5 (16.2)	58.9 (15.8)	40.0 (14.4)	51.4 (14.5)	51.0 (17.2)
**Selected physical conditions**	0.0 (0)	1.9 (1.2)	0.0 (0)	2.6 (1.7)	1.2 (1.5)
**Total number of conditions**	0.7 (0.8)	3.5 (2.0)	1.6 (1.2)	5.5 (2.7)	2.6 (2.5)
**Percent**					
**Age**					
18-29	23.7	6.6	31.8	9.9	15.3
30-44	29.1	12.6	32.2	22.8	21.8
45-59	26.0	25.4	25.4	36.0	27.3
60+	21.2	55.5	10.6	31.3	35.6
**Male gender**	56.8	47.9	41.9	37.5	49.3
**Race/ethnicity**					
White	78.4	82.8	83.5	80.5	80.8
Black or African American	10.6	8.9	5.9	8.1	9.3
American Indian or Alaskan Native	0.5	0.9	0.8	1.6	0.9
Asian	2.3	1.2	1.7	0.5	1.5
Other	4.1	3.2	5.5	6.0	4.1
Missing	4.1	3.0	2.5	3.3	3.4
**Education**					
Less than high school	6.5	9.3	7.6	10.7	8.3
High school	28.7	32.3	27.5	29.8	30.2
Some college	30.6	29.7	32.2	34.7	31.0
Bachelor's degree or higher	34.2	28.8	32.6	24.8	30.5
**Current employment status**					
Working	66.9	42.8	64.4	40.4	53.1
Not working (looking)	9.8	3.8	15.3	8.5	7.6
Retired	13.3	40.1	6.8	19.1	24.3
Not Working (not looking)	10.0	13.3	13.6	32.1	15.0
**Marital Status**					
Married/Living with partner	60.1	61.2	51.7	49.7	58.4
Widowed	3.2	10.4	0.8	7.0	6.5
Separated/Divorced	11.4	14.4	13.1	24.1	14.7
Never married	25.2	14.0	34.3	19.2	20.4

As also shown in Table 1, 63% of those in the study were at least 45 years of age and 36% at least age 60. This distribution is reflective of the intentional oversampling of individuals age 65 and older for the 2009 Normative Survey. However, this oversampling did not bias the regression results as all regression analyses were conducted using weights that adjusted the age and gender distributions of the study sample to the age and gender distributions of the 2009 US general population. A total of 1,505 participants were included in the *Healthy* group; 1,522 in the *Physical Health Condition*; 236 in the *Mental Health Condition*; and 614 in the *Physical and Mental Health Condition (Comorbidity)* groups.

### Reporting frequency of specific conditions

Overall, the average number of chronic conditions reported was 2.6 (SD = 2.5). The five most commonly reported were anxiety (n = 629, 16%), osteoarthritis and degenerative arthritis (n = 583, 15%), diabetes (n = 580, 15%), migraine (n = 574, 15%) and depression (n = 507, 13%). Table 2 provides a list of chronic conditions included in the present study, with corresponding frequency of self-report for both males and females.

**Table 2:** Percent of participants reporting each condition from the chronic condition checklist

	**Total sample**	**Males**	**Females**
**Body System**			
**Cardiovascular**			
heart attack in the last year	1.3	1.7	0.9
congestive heart failure (CHF)	3.5	4.0	3.1
angina or coronary artery disease	4.4	6.0	2.9
other heart conditions, such as problems with heart valves or the rhythm of your heartbeat	12.0	13.2	10.8
**Endocrine**			
diabetes or high blood sugar	15.0	16.9	13.1
**Respiratory**			
chronic obstructive pulmonary disease (COPD)	4.6	4.3	4.9
**Musculoskeletal**			
rheumatoid arthritis (RA)	8.2	7.9	8.4
osteoarthritis, degenerative arthritis	15.0	10.9	19.1
osteoporosis	6.6	2.3	10.7
**Gastrointestinal**			
stomach disease, such as gastritis or duodenitis	4.1	3.3	4.8
irritable bowel syndrome (IBS)	8.3	5.7	10.8
kidney disease	2.4	2.7	2.1
liver disease, such as Hepatitis B or C	2.3	3.0	1.6
**Other Physical Health**			
stroke	3.2	3.4	3.0
migraine headaches	14.9	7.5	21.9
chronic fatigue syndrome or fibromyalgia	4.4	1.7	6.9
cancer, except skin cancer	8.0	8.4	7.7
HIV or AIDS	0.5	0.8	0.2
**Mental Health**			
depression	13.1	9.0	17.0
anxiety	16.2	12.2	20.2
alcohol or other substance use disorder	2.7	4.0	1.3

Note: a) Hypertension, b) nasal allergies or allergic rhinitis, c) obesity, d) anemia, e) gastro-esophageal reflux disease (GERD), and f) sleep apnea, although included in the checklist, were excluded from analyses because they were not considered chronic conditions.

Considering all conditions included in the analysis, women reported having more conditions than did men (mean = 2.8 [SD = 2.7] for women and mean = 2.0 [SD = 2.1] for men). As expected, there were differences between males and females in the ordering of conditions by frequency with which they were reported. Migraine was more common in women (21.9%) than men (7.5%), as was anxiety (20.2% for women versus 12.2% for men), depression (17% for women versus 9% for men) and osteoporosis (10.7% versus 2.3%).

Of those with any chronic condition, approximately 41% (n = 972) reported having a single condition (physical or mental health), 23% (n = 548) reported having 2, 15% (n = 344) reported 3, and the remainder (21%; n = 508) had 4 or more conditions. Among those reporting a single condition, approximately 19% (n = 181) reported having 1 single mental health condition. For those with 2 conditions, 68% (n = 374) had 2 physical conditions, 9% (n = 48) had 2 mental health conditions. The remainder (23%) reported having 1 physical and 1 mental health condition.

### HRQL burden of chronic physical health conditions

Relative to participants in the *Healthy* group, those in the *Physical Health Condition* group showed substantial decrements in PCS scores. On average, males in the *Physical Health Condition* group scored 6.4 points lower (95% Confidence Interval (CI) [−7.3, -5.4]) on PCS than did their *Healthy* counterparts. Similarly, females in the *Physical Health Condition* group scored 7.5 points lower (95% CI [−8.6, -6.4]) than did females defined as *Healthy*. Relative to their PCS scores, decrements in MCS for the *Physical Health Condition* group were smaller for both males and females. Compared to scores for *Healthy* participants, MCS scores for males and females in the *Physical Health Condition* group were 1.7 points (95% CI [−2.7, -0.7]) and 2.1 points (95% CI [−3.2, -0.9]) lower, respectively. These results are shown in Table 3.

**Table 3:** Mean PCS and MCS decrements associated with a chronic physical health condition, a chronic mental health condition, or with co-morbid conditions (All compared to healthy reference group)

		**Males**		**Females**
	**Incremental decrement**	**Total decrement**	**Incremental decrement**	**Total decrement**
**PCS**				
≥ 1 Chronic **Physical Health** Condition **Only**	−6.4 (−7.3,-5.4)		−7.5 (−8.6,-6.4)	
**Addition** of ≥ 1 Chronic **Mental Health** Condition	−4.5 (−5.9,-3.2)	−10.9 (−12.3, 9.6)	−3.7 (−4.9,-2.5)	−11.2 (−12.5, -9.9)
≥ 1 Chronic **Mental Health** Condition **Only**	−0.7 (−2.6, 1.2)		0.3 (−2.5,3.1)	
**Addition** of ≥ 1 Chronic **Physical Health** Condition	−10.2 (−12.3,-8.1)	−10.9 (−12.3, 9.6)	−11.5 (−14.3,-8.6)	−11.2 (−12.5, -9.9)
**MCS**				
≥ 1 Chronic **Physical Health** Condition **Only**	−1.7 (−2.7,-0.7)		−2.1 (−3.2,-1.0)	
**Addition** of ≥ 1 Chronic **Mental Health** Condition	−13.6 (−15.0,-12.2)	−15.3 (−16.7, -14.0)	−13.1 (−14.3,-11.9)	−15.2 (−16.6, -14.1)
≥ 1 Chronic **Mental Health** Condition **Only**	−11.3 (−13.3,-9.3)		−11.9 (−14.7,-9.0)	
**Addition** of ≥ 1 Chronic **Physical Health** Condition	−4.0 (−6.2,-1.8)	−15.3 (−16.7, -14.0)	−3.3 (−6.2,-0.4)	−15.2 (−16.6, -14.1)

PCS = Physical Component Summary score.

MCS = Mental Component Summary score.

Values in parentheses are confidence intervals (CIs).

### HRQL burden of chronic mental health conditions

Not surprisingly, the MCS scores for participants in the *Mental Health Condition* group revealed large decrements compared to scores for the *Healthy* group. Decrements were similar for males (11.3 points lower; 95% CI [−13.3, -9.3]) and females (11.9 points lower; 95% CI [−14.7, -9.0]). For both males and females, PCS scores of those in the *Mental Health Condition* were not meaningfully different than PCS scores of those in the *Healthy* group. Scores were 0.7 points lower (95% CI [−2.6, 1.2]) for males and 0.3 points higher (95% CI [−2.5, 3.1]) for females (Refer again toTable 3).

### Physical health conditions: incremental HRQL burden of mental health comorbidity

Table 3 shows that for those in the *Physical Health Condition* group*,* a comorbid *Mental Health Condition* decreased PCS scores by an additional 4.5 points (95% CI [−5.9, -3.2]) (males) and 3.7 points (95% CI [−4.9, -2.5]) (females). Thus, total reductions were 10.9 and 11.2 points compared to the *Healthy* group. The incremental MCS burden of a mental health comorbidity to *Physical Health Conditions* alone was more pronounced, with increased decrements of −13.6 for males (95% CI = [−15.0, -12.2]) and −13.1 for females (95% [−14.3, -11.9]). For both males and females, total MCS burden was approximately 15 points when the comorbid group was compared to the *Healthy* group.

### Mental health conditions: incremental HRQL burden of physical health comorbidity

Finally, Table 3 shows that relative to participants in the *Mental Health Condition* group, those reporting both a *Mental Health* and a *Physical Health Condition* experienced lower HRQL as assessed by both PCS and MCS scores. PCS scores were 10.2 points lower (95% [−12.3, -8.1]) for males and 11.5 points lower (95% CI [−14.3, -8.6]) for females. Compared to those categorized as having a *Mental Health Condition* only, average additional decrements in MCS were 4.0 points (95% CI [−6.2, -1.8]) and 3.3 points (95% CI [−6.2, -0.4) (respectively) for males and females.

### HRQL burden across *physical disease clusters*

MCS decrements associated with a *Mental Health Condition* only (11.3 and 11.9 points, for males and females, respectively), along with the incremental effects for each of the 5 comorbid *Physical Disease Clusters.* Although comorbid gastrointestinal and musculoskeletal conditions were linked to the greatest overall MCS burden, the additional burden associated with a physical comorbidity was similar across each of the 5 clusters (3.6 to 5.6 points). Total MCS decrements were slightly larger for males than for females across the *Physical Disease Clusters* (data not shown).

Compared to the *Healthy* group, those in each *Physical Disease Cluster* showed PCS point reductions of 6.8 (gastrointestinal) to 11.7 (respiratory). Reductions were similar for males and females. The additional PCS reductions associated with a comorbid *Mental Health Condition* showed somewhat different patterns, depending on the particular *Physical Disease Cluster*. For 4 of 5 clusters, presence of a comorbid *Mental Health Condition* decreased PCS scores by approximately 5 additional points. For those with a cardiovascular condition however, a comorbid *Mental Health Condition* showed a smaller impact on PCS scores (−2.9 points). For each of the 5 Physical Disease Clusters, PCS decrements were almost identical for males and females (data not shown).

## Discussion

The goal of this study was to examine the burden of self-reported chronic *physical* and/or *mental health conditions* on each of the two commonly-used SF36v2 summary measures of HRQL; the Mental Component Summary (MCS) score and the Physical Component Summary (PCS) score. Consistent with findings of prior studies, the impact of at least 1 chronic *physical* condition for this sample of adults in the US general population was reflected to a greater extent in PCS rather than MCS scores [20,39]. Similarly, the impact of at least 1 chronic *mental health* condition was primarily reflected in MCS rather than PCS.

Relative to individuals with only *physical health* conditions, males and females reporting ≥ 1 comorbid *mental health* condition showed (on average) an additional PCS decrement of 4.5 and 3.7 points, respectively. These values are larger than 2-points, which is the recommended minimally important difference (MID) for group comparisons [35]. The finding that a comorbid chronic mental health condition was associated with an additional HRQL decrement is consistent with those reported in prior studies [15,17]. One of the strengths of the present study is that it provides quantitative information on specific PCS and MCS decrements associated with a comorbid condition related to mental health, as well as a comorbid condition related to physical health.

Compared to having a *mental health condition* only, the presence of ≥ 1 comorbid *physical health condition* led to further MCS decrements of 3.3 points for females and 4.0 points for males. Both values are above the recommended MID for MCS, which is 3-points [35]. The observed incremental impact of a physical comorbidity on MCS and the incremental impact of mental health comorbidity on PCS scores were robust across groups organized by ‘Physical Disease Clusters’. Total decrements in PCS scores associated with a chronic physical condition and a comorbid mental health condition were greater for the musculoskeletal, endocrine, and respiratory disease clusters (15.0 to 16.6 points) than for the gastrointestinal or cardiovascular disease clusters (11.7 to 12.0 points).

In the present study, overall patterns regarding HRQL decrements associated with physical and mental health comorbidities were similar for males and females. This finding differs from several other studies reporting lower HRQL scores for females with chronic physical or mental health conditions [22,37,40,41].

Current findings underscore the complexity of identifying and managing patients with multiple chronic conditions, and the importance of screening for- and treating conditions related to both physical and mental health. To illustrate, results of a recent randomized clinical trial showed that for older adults with arthritis and comorbid depression, improvements in depressive symptoms were associated not only with decreased arthritis-related pain, but with improved functional status and quality of life [7].

### Limitations

As with all studies, current results should be interpreted within the context of relevant limitations. Our approach for this set of analyses was a broad-based one; with several types of data aggregation. These include the use of chronic disease groupings, disease clusters, and PCS and MCS Summary scores. While appropriate for assessing the incremental HRQL burden associated with chronic physical and/ or mental health conditions, certain distinctions were not assessed. These would include the 8 individual SF-36 domains, the different diseases within a particular disease cluster (e.g., type 1 versus type 2 diabetes), and different levels of disease severity. While beyond the scope of this study, future efforts may benefit from additional, more fine-grained analyses of the normative data.

The present results were based exclusively on self-reported information, with no diagnostic assessment or clinical records to provide corroboration. However, self report of chronic conditions has demonstrated reasonable levels of sensitivity and specificity when compared to information obtained from medical chart review [42,43]. Participants in the *Healthy* group may have had undiagnosed conditions or those not included in the chronic condition checklist. Finally, those with certain very severe or disabling chronic conditions, or those without internet access may have been precluded from participating in a panel survey, and therefore could be underrepresented.

Several factors potentially important in understanding the relationship of chronic conditions and HRQL were not included in the present set of analyses. For example, analyses did not incorporate bio-psychosocial risk factors such as smoking, being overweight and/or sedentary, or lacking social support; all of which have been shown to correlate with greater impairments in HRQL for those with chronic conditions [21,24,30,44]. To the extent allowed by participant sample sizes, future analyses might also include subgroups based on age, race/ethnicity, or income [27,39,45]. However, research has shown the magnitude of HRQL decrements associated with two or more chronic conditions to be largely independent of socioeconomic factors [27].

## Conclusions

Results provide quantitative information for US adults on specific PCS and MCS score decrements associated with a comorbid condition related to mental health, as well as a comorbid condition related to physical health. Both PCS and MSC scores have shown to be significant predictors of outpatient and inpatient service utilization for individuals with chronic conditions such as arthritis. Thus, monitoring both aspects of functioning and well-being may lead to improved individual health outcomes and less use of costly healthcare services [46,47].

Note: A portion of this work was presented in poster format at the 16^th^ Annual International Meeting of the International Society for Pharmacoeconomics and Outcomes Research, May 21–25, Baltimore, MD.

## Competing interests

At the time of original submission, all authors were full-time employees of QualityMetric, part of OptumInsight Life Sciences, which publishes the SF-36v2® (the measure of HRQL for this paper).

## Authors’ contributions

All authors made substantive intellectual contributions to the study and/or the paper, and will take public responsibility for appropriate portions of its content. All have given final approval of the submitted version. MB: conception and design; revision of manuscript for important intellectual content. R R-B: conception and design; analysis and interpretation of data; revision of manuscript for important intellectual content. MKW: conception and design; revision of manuscript for important intellectual content. MM: draft of manuscript; revision of manuscript for important intellectual content. JB: conception and design; interpretation of data ST: draft of manuscript; revision of manuscript for important intellectual content. All authors read and approved the final manuscript.
